# Role of [^18^F]FAPI-04 in staging and therapeutic management of intrahepatic cholangiocarcinoma: prospective comparison with [^18^F]FDG PET/CT

**DOI:** 10.1186/s13550-024-01145-y

**Published:** 2024-09-11

**Authors:** Jiucen Liang, Shuqin Jiang, Jingjing Song, Danyang Chen, Shaojuan Weng, Shuyi Li, Hao Peng, Zhidong Liu, Jing Zhang, Yuanlin Chen, Songquan Rao, Haipeng Chen, Rusen Zhang, Hao Liu, Linqi Zhang

**Affiliations:** 1 Department of Nuclear Medicine, Affiliated Cancer Hospital & Institute of Guangzhou Medical University, 78 Hengzhigang Road, Guangzhou, Guangdong 510095 P.R. China; 2 Department of Pathology, Affiliated Cancer Hospital & Institute of Guangzhou Medical University, 78 Hengzhigang Road, Guangzhou, Guangdong 510095 P.R. China; 3 Tumor Research Institute, Affiliated Cancer Hospital & Institute of Guangzhou Medical University, 78 Hengzhigang Road, Guangzhou, Guangdong 510095 P.R. China

**Keywords:** [^18^F]FAPI-04, [^18^F]FDG, PET/CT, Intrahepatic cholangiocarcinoma, Cancer-associated fibroblasts

## Abstract

**Background:**

Fluorine-18 fluorodeoxyglucose ([^18^F]FDG) positron emission tomography/computed tomography (PET/CT) has some limitations in diagnosis of Intrahepatic cholangiocarcinoma (ICC).

**Materials and methods:**

Patients with histologically confirmed ICC who underwent both [^18^F]FDG and ^18^F-labeled fibroblast-activation protein inhibitors ([^18^F]FAPI)-04 PET/CT were prospectively analyzed. The maximum standard uptake value (SUV_max_), tumor-to-background ratio (TBR), metabolic tumor volume (MTV), total lesion glycolysis (TLG), [^18^F]FAPI–avid tumor volume (FTV), total lesion fibroblast activation protein expression (TLF) were compared between the two modalities by paired Wilcoxon signed-rank test and Mann–Whitney U test, and McNemar’s test was used to assess the diagnostic accuracy between the two techniques.

**Results:**

In total, 23 patients with 389 lesions were included. Compared to [^18^F]FDG, [^18^F]F-FAPI-04 PET/CT demonstrated a higher detection rate for intrahepatic lesions (86.3% vs. 78.2% *P* = 0.040), lymph node metastases (85.2% vs. 68.2%, *P* = 0.007), peritoneal metastases (100% vs. 93.8%), and bone metastases (100% vs. 70.5%, *P* < 0.001). [^18^F]FAPI-04 PET showed higher SUV_max_, TBR and greater tumor burden values than [^18^F]FDG PET in non-cholangitis intrahepatic lesions (SUV_max_: 8.7 vs. 6.4, *P* < 0.001; TBR: 8.0 vs. 3.5, *P* < 0.001; FTV vs. MTV: 41.3 vs. 12.4, *P* < 0.001; TLF vs. TLG: 223.5 vs. 57.0, *P* < 0.001), lymph node metastases (SUV_max_: 6.5 vs. 5.5, *P* = 0.042; TBR: 5.4 vs. 3.9, *P* < 0.001; FTV vs. MTV: 2.0 vs. 1.5, *P* = 0.026; TLF vs. TLG: 9.0 vs. 7.8 *P* = 0.024), and bone metastases (SUV_max_: 9.7 vs. 5.25, *P* < 0.001; TBR: 10.8 vs. 3.0, *P* < 0.001; TLF vs. TLG: 9.8 vs. 4.2, *P* < 0.001). However, [^18^F]FDG showed higher radiotracer uptake (SUV_max_: 14.7 vs. 8.4, *P* < 0.001; TBR: 7.4 vs. 2.8, *P* < 0.001) than [^18^F]FAPI-04 PET/CT for 6 patients with obstructive cholangitis. [^18^F]FAPI-04 PET/CT yielded a change in planned therapy in 6 of 23 (26.1%) patients compared with [^18^F]FDG.

**Conclusions:**

[^18^F]FAPI-04 PET/CT had higher detection rate and radiotracer uptake than [^18^F]FDG PET/CT in intrahepatic lesions, lymph node metastases, and distant metastases, especially in bone. Therefore, [^18^F]FAPI-04 PET/CT may be a promising technique for diagnosis and staging of ICC.

**Trial registration:**

Clinical Trials, NCT05485792. Registered 1 August 2022, retrospectively registered, https//clinicaltrials.gov/study/NCT05485792?cond=NCT05485792&rank=1.

**Supplementary Information:**

The online version contains supplementary material available at 10.1186/s13550-024-01145-y.

## Introduction

Intrahepatic cholangiocarcinoma (ICC) and extrahepatic cholangiocarcinoma are collectively the second most common types of primary liver cancer, ICC arises from the epithelial cells of the intrahepatic bile ducts and accounting for only 3% of gastrointestinal malignancies [1]. Diagnosis often occurs at an advanced stage, resulting in only 22% of patients eligible for surgery [2, 3]. Accurate early diagnosis and staging are vital for determining appropriate treatments to improve patient outcomes. Traditional imaging modalities such as contrast computed tomography (CT) and magnetic resonance imaging (MRI) have limitations, especially in detecting lymph node involvement and distant metastasis [4–6]. Although Fluorine-18 fluorodeoxyglucose ([^18^F]FDG) positron emission tomography/computed tomography (PET/CT) is valuable in many cancers for TNM staging and response assessment, it faces challenges in ICC due to false negatives caused by variable [^18^F]FDG uptake and physiological liver activity, as well as low sensitivity of lymph node staging and interference from gastrointestinal uptake [7–11]. Therefore, a more effective PET tracer for ICC is needed.

ICC is typically associated with a dense desmoplastic stroma in cancer-associated fibroblasts (CAFs), which foster tumor growth, invasion, and resistance to therapy [12, 13]. Fibroblast activation protein (FAP) is highly expressed in CAFs of many epithelial carcinomas while has limited expression in normal tissue, making it a promising target for ICC imaging [14]. Previous studies have demonstrated that ^68^Ga-labeled fibroblast activating protein inhibitor ([^68^Ga]Ga-FAPI) PET/CT exhibits a higher maximum standardized uptake value (SUV_max_) and tumor-to-background ratio (TBR) and detects more lesions in ICC compared to [^18^F]FDG, but these studies had small sample sizes and included recurrent cases [10, 15–19]. Recent studies also have shown that [^68^Ga]Ga-FAPI PET/CT measures significantly larger gross tumor volumes in different tumors compared to [^18^F]FDG-PET/CT, and an accurate tumor delineation, thereby reinforcing its utility in pretherapeutic staging [20]. In addition, a recently published study highlights the potential of volumetric indices on baseline [^68^Ga]Ga-FAPI PET/CT for predicting treatment response in patients with hepatocellular carcinoma [21]. However, to the best of our knowledge, semiquantitative indices such as metabolic tumor volume (MTV), However, total lesion glycolysis (TLG), [^18^F]FAPI–avid tumor volume (FTV), and total lesion fibroblast activation protein expression (TLF) in ICC have not yet been reported. Moreover, the short half-life and insufficient production of ^68^Ga restricts the use of [^68^Ga]Ga-FAPI [22]. Recently, several ^18^F-labeled FAPI tracers have been evaluated in some malignancies and exhibited good performance for detecting malignant tumors [23]. Our previous work indicated that [^18^F]FAPI-04 PET/CT has high sensitivity in detecting liver malignancies with [^18^F]FDG non-avidity, including ICC [10]. The impressive diagnostic performance of [^18^F]FAPI-04 PET/CT encouraged us to explore its clinical utility in primary staging of ICC.

Therefore, the aim of this prospective study was to assess the potential value of [^18^F]FAPI-04 PET/CT in staging and treatment management of ICC patients, and compare the results with those of [^18^F]FDG PET/CT.

## Materials and methods

### Patients

This prospective study was authorized by the ethics committee of our hospital (ethics committee permission No.2021-sw07; clinical trial registration: NCT05485792). From March 2021 and June 2023, a total of 162 patients with liver tumor were considered as candidate participants. The enrolled patients met the following criteria: (i) patients with newly diagnosed ICC based on histopathology; (ii) age ≧ 18 years old; and (iii) patients who agreed to receive paired [^18^F]FDG PET/CT and [^18^F]FAPI-04 PET/CT scans within one week. The exclusion criteria were as follows: (i) patients who have received chemotherapy, radiation therapy, or targeted therapy prior to PET scanning; (ii) patients who had another primary cancer at the time of evaluation; and (iii) incapacity or reluctant to provide informed consent (signed by the participant, parent, or legal representative).

### Synthesis of radiopharmaceuticals

[^18^F]FDG was automatically synthesized using a PET trace cyclotron (GE Healthcare) and the [^18^F]FDG synthesizer module Tracerlab FXF-N (Beijing PET Biotechnology Co. Ltd). According to the following procedure, ^18^F-labeled FAP tracers were generated by adding ^18^F-eluent (37–74 GBq) to a solution of DOTA-FAPI (80 nmol) in 2.0 mol/L NaOAc aqueous (1 mL). After being heated at 105 °C for 15 min, the reaction mixture was purified using a straightforward solid-phase extraction method followed by cartridge separation. Analysis and quality control of the prepared products were performed in an analytical C18 HPLC column (radiochemical purity > 95%).

### [^18^F]FDG/[^18^F]FAPI-04 PET/CT acquisition and imaging

[^18^F]FDG and [^18^F]FAPI-04 PET/CT were performed using a PET/CT scanner (Discovery 710, GE Healthcare, Milwaukee, WI, USA) within 1 week. For [^18^F]FDG PET/CT scanning, patients fasted at least 6 h, maintaining venous blood glucose levels under 11.1 mmol/L prior to [^18^F]FDG administration. However, this was not necessary for [^18^F]FAPI-04 PET/CT scanning. The intravenous injection activity of [^18^F]FDG was calculated according to the patient’s weight (0.1mCi/kg). The intravenous injection activity of [^18^F]FAPI-04 was within 148–259 MBq. [^18^F]FDG PET images were acquired at approximately 60 min after injection, and [^18^F]FAPI-04 PET images were acquired at approximately 20 min after injection. All obtained data were transferred to the Advantage Workstation (version AW 4.7, GE Healthcare, Milwaukee, WI, USA) and reconstructed using the Bayesian penalized likelihood reconstruction algorithm (Q.clear, GE Healthcare, Milwaukee, WI, USA) with a penalization factor (beta) of 500.

### [^18^F]FDG and [^18^F]FAPI-04 PET/CT image analysis

All images were visually interpreted independently by two board certified nuclear medicine physicians who were blinded to the results of contrast-enhanced CT/MRI (ce-CT/MRI), pathology, any other PET/CT scan information, and the final diagnosis. The image was interpreted using the frame-to-frame comparison mode on the dual-image comparison format provided by the Advantage workstation, included visual analysis and quantitative assessment.

For visual analysis, lesions were divided into intrahepatic tumor and extrahepatic organs/regions metastasis (lymph nodes and distant metastasis) based on their location. Any focal accumulations of [^18^F]FDG and [^18^F]FAPI-04 that were higher than the surrounding background, were interpreted as a positive lesion, which was considered a suspected malignant lesion.

For semiquantitative analysis of the PET/CT data was performed on the Advantage Workstation (version AW 4.7, GE Healthcare, Milwaukee, WI, USA), a region of interest (ROI) was drawn along the entire lesion on the axial PET image or anatomical information presented by CT (lesions with low or equal tracer uptake). CT images were also used as reference to ensure the standardized uptake value (SUV) were measured from the same lesions from two different image sets. The maximum standardized uptake value (SUV_max_), the diameter and amount of lesions in per region were recorded. The tumor-to-background ratio (TBR) of each lesion was calculated by dividing the SUV_max_ of the lesion by the SUV_mean_ of the background tissue (liver background for liver lesions; mediastinal blood pool background for lymph nodes, pleura and peritoneal lesions; lung background for lung lesions; contralateral adrenal gland background for adrenal gland lesions; and L5 background for bone lesions). Furthermore, for these measurements such as metabolic tumor volume (MTV), total lesion glycolysis (TLG), [^18^F]FAPI–avid tumor volume (FTV) and total lesion FAP expression (TLF), a cuboid volume of interest (VOI) was drawn to include all focal lesions in each scan, with the tumor contours semi-automatically segmented using an SUV cutoff of 2.5. These contours were then manually checked and adjusted to exclude physiological or inflammatory uptake. The values of MTV, TLG, FTV, and TLF were then automatically generated from the final volumetric extraction.

In the visual comparative system for each lesion category, the visibility (meaning lesions can be easily observed on PET images, assessed by TBR contrast) or area of lesions detected by [^18^F]FAPI PET/CT was greater than that detected by [^18^F]FDG, the result was classified as “FAPI superior” and vice versa. When the visibility or area of lesions detected by both imaging modalities was the same, the result was classified as “equal”.

### Reference standard

All patients were confirmed ICC based on histological evaluation of biopsy or surgical specimens. Due to technical and ethical issues, histopathologic confirmation was not possible for all lesions, especially for nodal and distant metastases. Thus, a combination of clinical and multimodality radiographic follow-up for more than 6 months was taken as the reference standard of diagnosis, including ce-CT/MRI, abdominal ultrasound, whole-body bone scan, and whole-body PET/CT. Lesions were considered malignant during follow-up based on (i) lesions exhibiting typical malignant characteristics in multimodality medical imaging, including the lesions that were missing or insufficiently detected by [^18^F]FDG PET and [^18^F]FAPI-04 PET but for which ce-CT/MRI could interpreted; (ii) on radiological follow-up, lesions showed an increase in size/in [^18^F]FAPI-04 uptake, and/or a significant reduction in size after anticancer treatment. The TNM stage was assigned based on the eighth edition of the American Joint Committee on Cancer staging system [24].

### Statistical analysis

Quantitative variables are presented as the median [range (minimum to maximum)], and categorical variables are presented as frequencies (percentages). The diagnostic performance for ICC of [^18^F]FDG PET/CT and [^18^F]FAPI-04 PET/CT was compared using the McNemar’s test. The SUV_max_, TBR, MTV, TLG, FTV and TLF obtained from [^18^F]FDG and [^18^F]FAPI-04 PET images were compared using the paired Wilcoxon signed-rank test and Mann–Whitney U test. To assess the inter-reader agreement for [^18^F]FDG PET/CT and [^18^F]FAPI-04 PET/CT, Cohen’s kappa (κ) coefficients were calculated and expressed as linear-weighted values with 95% confidence intervals. The κ value interpretations are as follows: κ ≤ 0.20 indicates slight agreement; 0.21–0.40, fair; 0.41–0.60, moderate; 0.61–0.80, substantial; and 0.81–1.00, almost perfect. All statistical tests were performed using SPSS Statistics 22.0 (SPSS Inc., Chicago, IL, USA) software. *P* < 0.05 was considered statistically significant.

## Results

### Patients characteristics

**Fig. 1:**
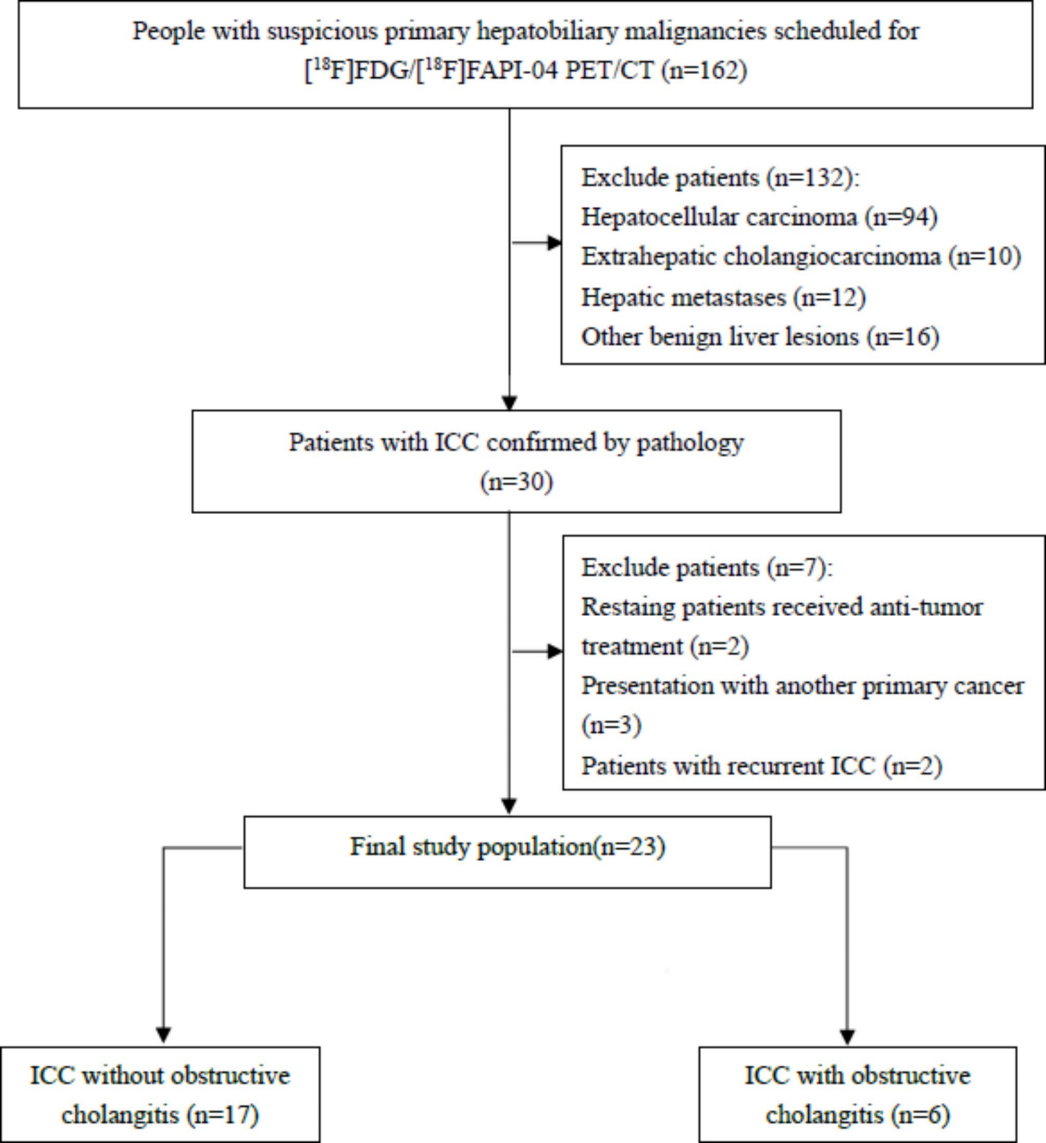
Study flowchart shows inclusion and exclusion criteria ICC = intrahepatic cholangiocarcinoma; ^18^F = fluorine 18; FAPI = fibroblast activation protein inhibitor; FDG = fluorodeoxyglucose; PET/CT = positron emission tomography/computed tomography

Between March 2021 and June 2023, 162 patients with suspected primary hepatobiliary malignancies underwent paired [^18^F]FDG and [^18^F]FAPI-04 PET/CT scans. Among them, 132 patients were excluded due to their final diagnoses confirmed by pathological examination: hepatocellular carcinoma (94), extrahepatic cholangiocarcinoma (10), hepatic metastases (12), and other benign liver lesions (16). Additionally, 7 patients with pathology-confirmed ICC were excluded due to anti-tumor treatment (2), the presence of another primary cancer (3), or recurrent ICC (2). As a result, a total of 23 patients with histological proven ICC (17 men and 6 women; median age, 61 [range, 39–71] years old) were finally enrolled. The flow chart was presented in Fig. 1. Among them, 26.1% (6 of 23) of patients were accompanied with obstructive cholangitis, and 56.5% (13 of 25) of patients were identified to had extrahepatic lesions (13 patients with lymph node metastasis and 6 patients with distant metastasis) (Table 1). According the eighth edition of the American Joint Committee on Cancer staging system, 8 patients were classified as clinical stage II, 9 patients as clinical stage III, and 6 patients as clinical stage IV.

**Table 1:** Baseline patient characteristics of the included patients

Description of patients	Number (23)
Age [years, median (IQR)]	61(39–71)
>60	11(47.8%)
≤ 60	12(52.2%)
Gender	
Male	17(73.9%)
Female	6(26.1%)
Obstructive cholangitis	6/23(26.1%)
Clinical biochemical testing	
AFP(> 20ng/ml)	2(8.7%)
CEA(> 5U/ml)	8(34.8%)
CA19-9(> 37U/ml)	17(73.9%)
Tumor number	
Solitary	12(52.2%)
Multifocal	11(47.8%)
Macrovascular invasion	7(30.4%)
Clinical stage/final diagnosis	
Stage I	0
Stage II	8(34.8%)
Stage III	9(39.1%)
Stage IV	6(26.1%)
Extrahepatic Lesions	
Lymph node metastasis	13(56.5%)
Distant metastasis	6(26.1%)

IQR = interquartile range; AFP = alpha-fetoprotein; CEA = carcinoembryonic antigen; CA 19 − 9 = carbohydrate antigen199

### Inter-reader agreement

According to Cohen’s kappa (κ), there was almost perfect inter-reader agreement between the two independent readers for both [^18^F]FDG PET/CT (κ = 0.805, *p* < 0.001) and [^18^F]FAPI-04 PET/CT (κ = 0.863, *p* < 0.001).

### [^18^F]FDG and [^18^F]FAPI-04 PET/CT in detection of intrahepatic lesions

In the final cohort of 23 patients, [^18^F]FAPI-04 PET/CT demonstrated greater sensitivity (100% vs. 87.0%) in identifying primary ICC tumors compared to [^18^F]FDG PET/CT, based on histological evaluations of biopsy or surgical specimens (Table 2). To assess the diagnostic accuracy of intrahepatic metastasis, a total of 111 suspected intrahepatic metastases (101 metastases, 8 hemangiomas and 2 inflammatory lesions with the final diagnosis) were analyzed, [^18^F]FDG PET/CT showed no or slight uptake in all 10 lesions, while the 2 inflammatory lesions exhibited increased [^18^F]FAPI-04 uptake causing 2 false-positive findings. The lesion-based sensitivity, specificity, and accuracy of [^18^F]FDG PET/CT were 76.2% (77/101), 100% (10/ 10), and 78.4% (87/111), respectively, and 85.1% (86/101), 80.0% (8/10), and 84.7% (94/111) for [^18^F]FAPI-04 PET/CT (Table 2).

**Table 2:** Diagnostic performances of [^18^F]FDG and [^18^F]FAPI-04 PET/CT for evaluation of ICC primary and metastatic lesions

Parameters	[^18^F]FDG PET/CT			[^18^F]FAPI-04 PET/CT		
	Sensitivity (%)	Specificity (%)	Accuracy (%)	Sensitivity (%)	Specificity (%)	Accuracy (%)
Primary tumor	87.0% (20/23)	NA	NA	100% (23/23)	NA	NA
Intrahepatic metastasis	76.2% (77/101)	100% (10/10)	78.4% (87/111)	85.1% (86/101)	80.0% (8/10)	84.7% (94/111)
Lymph nodes metastasis	68.2% (60/88)	42.4% (14/33)	61.2% (74/121)	85.2% (75/88)	81.8% (27/33)	84.3% (102/121)
Distant metastasis	85.5% (100/117)	64.7% (11/17)	82.8% (111/134)	96.6% (113/117)	58.8% (10/17)	91.8% (123/134)
Bone metastasis	70.5% (31/44)	69.2% (9/13)	70.2% (40/57)	100% (44/44)	46.2% (6/13)	87.7% (50/57)
Other distant metastasis^†^	94.5% (69/73)	50.0% (2/4)	92.2% (71/77)	94.5% (69/73)	100% (4/4)	94.8% (73/77)

*Note* Numbers in parentheses are the numbers of lesions used to calculate the percentage

Other distant metastasis^†^ includes lung, pleura, peritoneal and adrenal metastases, excluding bone metastases. NA = not available

**Fig. 2:**
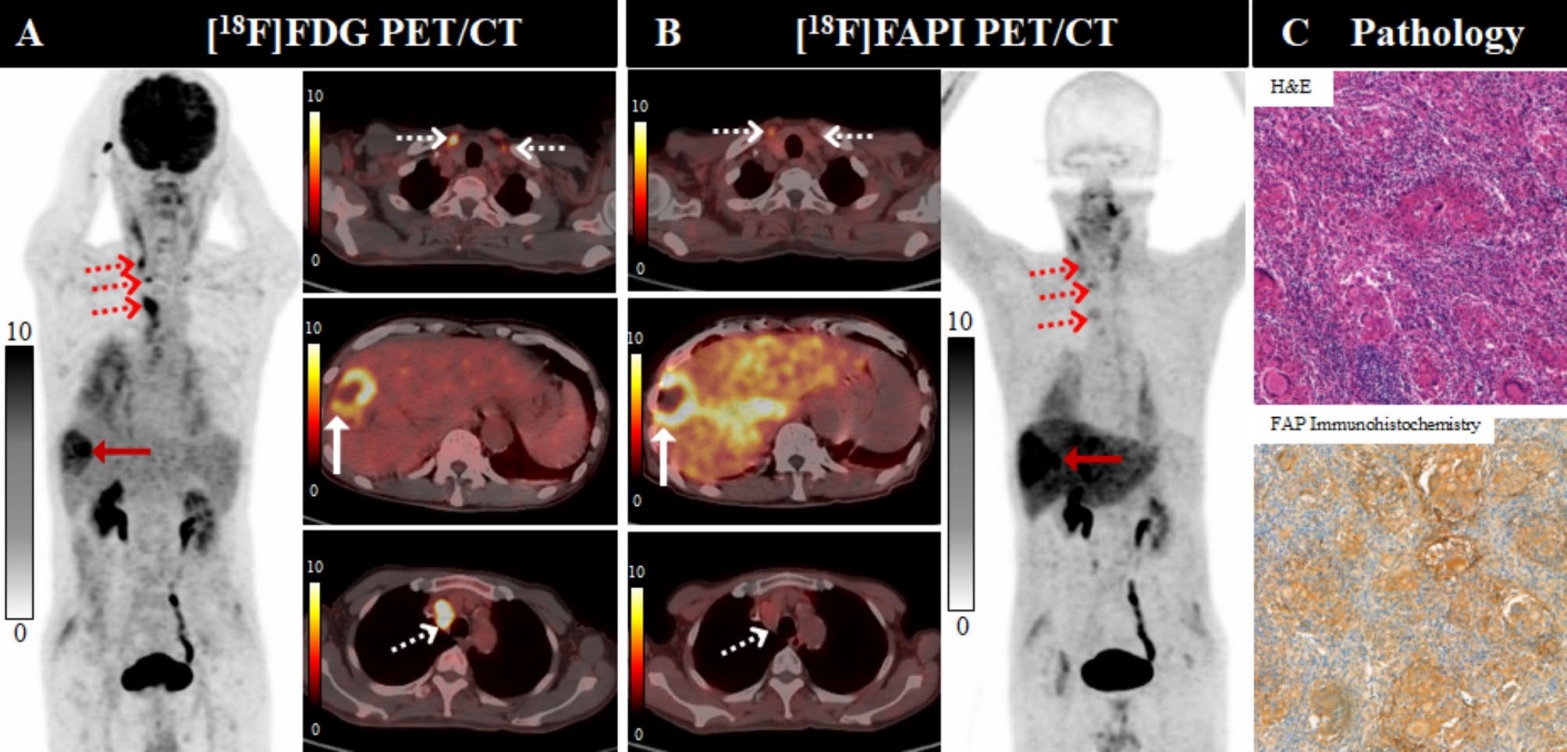
A 70-year-old male patient (Patient No. 19) with intrahepatic cholangiocarcinoma (ICC) was confirmed by biopsy. Compared with [^18^F]FDG PET/CT (**A**), [^18^F]FAPI-04 PET/CT (**B**) showed significantly increased radiotracer uptake in the primary tumor (white arrow in axial images), yet displayed elevated hepatic [^18^F]FAPI-04 activity in the liver parenchyma background. There were several lymph nodes (dotted arrows), follow-up evaluations confirmed reactive. Pathological findings (**C**) indicate a heightened expression for FAP

Among the 23 patients, 6 (26.1%) presented with obstructive cholangitis, intense [^18^F]FAPI-04 uptake was observed throughout the liver. The hepatic activity of [^18^F]FAPI-04 in the liver parenchyma background of patients with obstructive cholangitis (SUV_mean_=2.7 ± 0.87) was significantly higher than that of patients without obstructive cholangitis (SUV_mean_=0.95 ± 0.33;*P* < 0.001). There was no significant difference in the hepatic activity of [^18^F]FDG in the liver parenchyma background of patients with and without obstructive cholangitis (SUV_mean_: 2.11 ± 0.40 and 2.14 ± 0.45; *P* = 0.815). Contrastingly, the TBR of [^18^F]FAPI-04 in patients without obstructive cholangitis was significantly higher than that in patients with obstructive cholangitis (*P* = 0.004), and the representative images are presented in Fig. 2 and Supplemental Fig. 1.

**Fig. 3:**
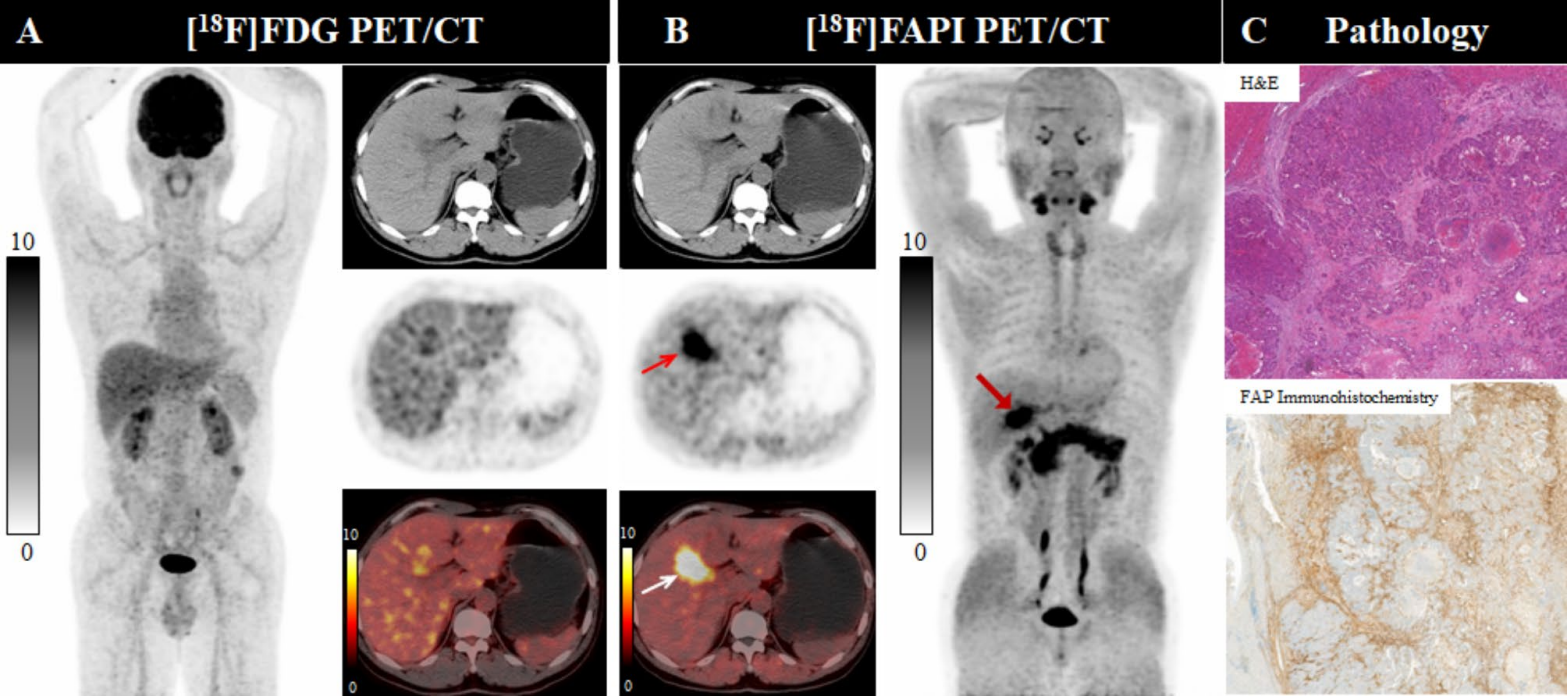
A 57-year-old male patient (Patient No. 4) with intrahepatic cholangiocarcinoma (ICC) was confirmed by postoperative pathology. [^18^F]FDG PET/CT (**A**) displayed no uptake in this lesion, conversely, [^18^F]FAPI-04 PET/CT (**B**) revealed intense uptake (SUV_max_ 13.7; TBR 11.4) in the hepatic segment IV/V lesion, as shown on both MIP images (large arrow) and axial images (small arrow). Pathological findings (**C**) indicate a heightened expression for FAP

Regarding the 17 ICC patients without obstructive cholangitis (17 primary tumors, and 70 intrahepatic metastases), [^18^F]FAPI-04 PET/CT visualized more primary tumors (100%, 17/17) than that of [^18^F]FDG PET/CT (82.4%, 14/17), as confirmed histopathologically. [^18^F]FAPI-04 PET/CT showed more intense uptake of the tracer by the primary tumors and had a higher TBR and a greater tumor burden than [^18^F]FDG PET/CT (SUV_max_, 14.0 vs. 9.9, *P* = 0.004; median TBR, 17.9 vs. 4.6, *P* = 0.001; FTV vs. MTV, 103.0 vs. 61.7, *P* < 0.001; TLF vs. TLG, 922.3 vs. 395.5, *P* < 0.001. Table 3; Fig. 3). In the semiquantitative analysis of 70 intrahepatic metastases, [^18^F]FAPI-04 PET/CT showed higher activity (median SUV_max_, 8.25 vs. 5.9, *P* < 0.001), clearer tumor delineation (median TBR, 6.0 vs. 3.1, *P* < 0.001) and greater tumor burden (median FTV vs. median MTV, 7.3 vs. 4.4, *P* = 0.005; median TLF vs. median TLG, 42.9 vs. 20.7, *P* = 0.004. Table 3) than [^18^F]FDG. For the 6 ICC patients with obstructive cholangitis (6 primary tumors, and 31 intrahepatic metastases), [^18^F]FAPI-04 and [^18^F]FDG PET/CT both visualized all the primary tumor (100%, 6/6), as confirmed histopathologically. [^18^F]FDG PET/CT showed more intense uptake of the tracer by the primary tumors, had a higher TBR than [^18^F]FAPI-04 PET/CT (median SUV_max_, 19.8 vs. 13.7; median TBR, 11.9 vs. 8.1, but both without statistical significance; Table 3). In the semiquantitative analysis of 31 intrahepatic metastases, [^18^F]FDG PET/CT also showed higher activity (median SUV_max_, 14.1 vs. 5.8, *P* < 0.001) and clearer tumor delineation (median TBR, 6.6 vs. 2.5, *P* < 0.001) than [^18^F]FAPI-04 PET/CT.

**Table 3:** Comparison of [^18^F]FDG PET/CT and [^18^F]FAPI-04 PET/CT for the intrahepatic and extrahepatic lesions of 23 patients

Description of lesions	No.of lesions	[^18^F]FDG PET/CT				[^18^F]FAPI-04 PET/CT				*P* value (FDG vs. FAPI)				
		Median SUV_max_ (range)	Median TBR (range)	Median MTV (cm^3^, range)	Median TLG (SUV_bw_.cm^3^, range)	Median SUV_max_ (range)	Median TBR (range)	Median FTV (cm^3^, range)	Median TLF (SUV_bw_.cm^3^, range)		SUV_max_	TBR	M/FTV	TLG/ F
Intrahepatic lesions	124	8.0 (1.6–46.5)	4.5 (0.7–21.1)	5.6 (0.5–710.0)	30.8 (2.0-4765.7)	8.6 (2.1–31.8)	6.5 (1.3–63.6)	8.2 (0.3–875.0)	58.3 (1.3-6405.7)		0.945	**0.007**	**< 0.001**	**< 0.001**
ICC without obstructive cholangitis	87	6.4 (1.6–20.9)	3.5 (0.7–17.4)	12.4 (0.5–710.0)	57.0 (2.0-3663.5)	8.7 (2.1–31.8)	8.0 (1.3–63.6)	41.3 (0.6–875.0)	223.5 (3.2-6405.7)		**< 0.001**	**< 0.001**	**< 0.001**	**< 0.001**
Primary tumor	17	9.9 (4.0-20.9)	4.6 (1.7–17.4)	61.7 (2.2–710.0)	395.5 (14.4-3663.5)	14.0 (4.3–31.8)	17.9 (3.6–63.6)	103.0 (14.4–875.0)	922.3 (80.9-6405.7)		**0.004**	**0.001**	**< 0.001**	**< 0.001**
Intrahepatic metastases	70	5.9 (1.6–19.6)	3.1 (0.7–16.3)	4.4 (0.5–41.9)	20.7 (2.0-177.6)	8.25 (2.1–15.0)	6.0 (1.3–21.4)	7.3 (0.6–61.5)	42.9 (3.2-394.3)		**< 0.001**	**< 0.001**	**0.005**	**0.004**
ICC with obstructive	37	14.7 (3.5–46.5)	7.4 (1.8–21.1)	5.9 (0.6–264.0)	58.6 (2.2-4765.7)	8.4 (3.4–16.9)	2.8 (1.4–27.5)	4.9 (0.3–221.0)	29.6 (1.3–2168.0)		**< 0.001**	**< 0.001**	0.968	0.494
Primary tumor	6	19.8 (7.8–46.5)	11.9 (3.1–21.1)	72.3 (10.2–264.0)	502.5 (92.1-4765.7)	13.7 (10.1–16.9)	8.1 (2.5–27.5)	77.3 (19.1–221.0)	686.0 (144.5–2168.0)		0.116	0.917	0.345	0.917
Intrahepatic metastases	31	14.1 (3.5–40.6)	6.6 (1.8–18.5)	2.4 (0.6–15.2)	22.9 (2.2-383.5)	5.8 (3.4–13.6)	2.5 (1.4–9.1)	2.9 (0.3–15.2)	17.9 (1.3-125.4)		**< 0.001**	**< 0.001**	0.196	0.087
Extrahepatic lesions	205	5.3 (1.3–19.2)	3.8 (0.9–38.4)	1.4 (0.1–33.6)	5.3 (0.3-284.5)	5.7 (0.9–18.9)	6.35 (0.7–22.0)	1.6 (0.1–28.1)	7.5 (0.3-182.4)		**0.001**	**< 0.001**	**0.004**	**< 0.001**
Lymph node metastasis	88	5.5 (1.4–17.4)	3.9 (0.9–13.4)	1.5 (0.1–33.6)	7.8 (0.3-284.5)	6.5 (1.4–13.5)	5.4 (0.7–20.2)	2.0 (0.2–28.1)	9.0 (0.8-180.9)		**0.042**	**< 0.001**	**0.026**	**0.024**
Distant metastasis	117	5.1 (1.3–19.2)	3.7 (0.9–38.4)	1.3 (0.1–32.4)	4.4 (0.3-176.5)	4.9 (0.9–18.9)	7.2 (1.2–22.0)	1.5 (0.1–30.4)	5.9 (0.3-205.2)		0.059	**0.011**	0.066	**< 0.001**
Bone metastases	44	5.25 (1.7–14.9)	3.0 (0.9–7.4)	1.7 (0.1–32.4)	4.2 (0.3-176.5)	9.7 (2.7–18.9)	10.8 (3.0–22.0)	1.7 (0.3–30.4)	9.8 (1.6-205.2)		**< 0.001**	**< 0.001**	0.383	**< 0.001**
Peritoneal metastases	64	5.45 (1.4–19.2)	6.3 (1.7–38.4)	1.3 (0.5–11.5)	5.3 (1.2–69.3)	4.45 (1.8–11.2)	6.8 (2.3–17.3)	1.6 (0.5–20.0)	5.9 (1.4–74.1)		**0.003**	0.126	**0.025**	0.757
Pleural metastasis	4	2.8 (2.4–4.1)	3.1 (2.7–4.6)	0.6 (0.5–1.6)	1.2 (1.1-2.0)	3.05 (1.8–3.3)	2.5 (1.5–2.8)	0.3 (0.1–1.4)	0.7 (0.3–2.9)		-	-	-	-
Lung metastasis	4	1.5 (1.3–2.2)	5.0 (4.3–7.3)	0.8 (0.2–1.1)	1.4 (0.8–2.1)	1.0 (0.9–1.9)	1.7 (1.5–3.2)	0.4 (NA)	1.25 (NA)		-	-	-	-
Adrenal metastasis	1	7.3 (NA)	2.9 (NA)	3.1 (NA)	14.0 (NA)	2.7 (NA)	1.3 (NA)	0.2 (NA)	0.52 (NA)		-	-	-	-

*Note* Bold fonts indicate significant difference between FDG and FAPI (*P* < 0.05)

ICC = intrahepatic cholangiocarcinoma; SUV_max_ = maximum standardized uptake value; TBR = Tumor-to-background ratio; SUV_bw_ = SUV_body weight_; MTV = metabolic tumor volume; TLG = total lesion glycolysis; FTV = [^18^F]FAPI–avid tumor volume; TLF = total lesion FAP expression; NA = not available

### [^18^F]FDG and [^18^F]FAPI-04 PET/CT for assessment of lymph node metastasis

According to the visual analysis for lymph node metastasis, a total of 121 suspected lymph nodes (88 metastatic lymph node lesions and 33 reactive lymph nodes with the final diagnosis) in 23 ICC patients were evaluated. [^18^F]FDG PET/CT imaging demonstrated increased uptake in 19 reactive lymph nodes with false-positive judgment for lymph node assessment. Conversely, the majority of reactive lymph nodes demonstrated low or absent radiotracer uptake on [^18^F]FAPI-04 PET/CT imaging, with only 6 false-positive findings. The sensitivity, specificity and accuracy of [^18^F]FDG PET/CT were slightly lower than [^18^F]FAPI-04 PET/CT for lymph node evaluation. (68.2% [60/88] vs. 85.2% [75/88]), *P* = 0.007; 42.4% [14/33] vs. 81.8% [27/33], *P* < 0.001; 61.2% [74/121] vs. 84.3% [102/121], *P* < 0.001. Table 2). The SUV_max_ (6.5 vs. 5.5, *P* = 0.042), TBR (5.4 vs. 3.9, *P* < 0.001), and tumor burden (median FTV vs. median MTV: 2.0 vs. 1.5, *P* = 0.026; median TLF vs. median TLG: 9.0 vs. 7.8, *P* = 0.024.) of [^18^F]FAPI-04 PET in lymph node metastasis were significantly higher and greater than that in [^18^F]FDG PET (Table 3).

### [^18^F]FDG and [^18^F]FAPI-04 PET/CT in evaluation of distant metastasis

To assess the diagnostic accuracy of distant metastases, 134 suspicious metastatic lesions from 23 ICC patients were evaluated. Of these, 117 lesions were identified as metastatic lesions in 6 patients based on the reference standards, including 44 bone metastases, 64 peritoneal metastases, 4 pleura metastases, 4 lung metastases and 1 adrenal gland metastasis. [^18^F]FAPI-04 PET had greater sensitivity (96.6% [113/117] vs. 85.5% [100/117], *P* = 0.006) and accuracy (91.8% [123/134] vs. 82.8% [111/134], *P* = 0.028 ) than [^18^F]FDG PET. The specificity of PET using [^18^F]FAPI-04 was lower than that of [^18^F]FDG (58.8% [10/17] vs. 64.7% [11/17]) because more false-positive lesions were observed on [^18^F]FAPI-04 PET (including fracture [*n* = 2], osteofibrous dysplasia [*n* = 2], degenerative osteophyte [*n* = 3]), but it was not significantly different (*P* = 0.724).

**Fig. 4:**
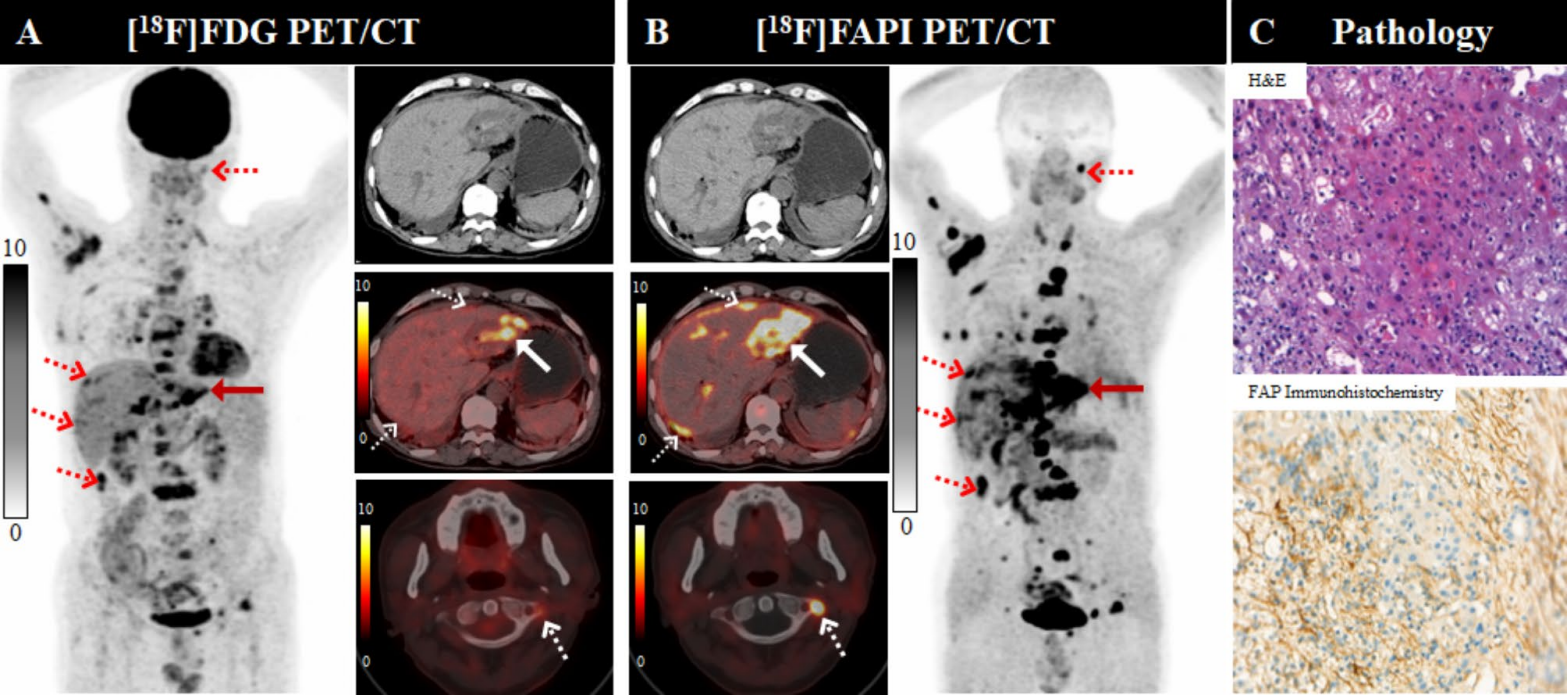
A 61-year-old male patient (Patient No. 16) with intrahepatic cholangiocarcinoma (ICC) was confirmed by biopsy. Compared with [^18^F]FDG PET/CT) (**A**), [^18^F]FAPI-04 PET/CT (**B**) demonstrated higher radiotracer uptake in the primary tumor ( SUV_max_ of 13.7, solid arrow), as well as in peritoneal and bone metastatic lesions (dotted arrows). Pathological findings (**C**) indicate a heightened expression for FAP

Regarding the 44 bone metastases, [^18^F]FAPI-04 PET had a significant higher sensitivity and accuracy than [^18^F]FDG PET (100% [44/44] vs. 70.5% [31/44], *P* < 0.001; 87.7% [50/57] vs. 70.2% [40/57], *P =* 0.022) (Table 2) and [^18^F]FAPI-04 PET showed higher SUV_max_, TBR and larger tumor burdens than [^18^F]FDG PET in bone metastases evaluation (median SUV_max_: 9.7 vs. 5.25, *P* < 0.001; median TBR: 10.8 vs. 3.0, *P* < 0.001; median TLF vs. median TLG: 9.8 vs. 4.2, *P* < 0.001) (Table 3; Fig. 4). [^18^F]FDG PET/CT showed 4 false positive uptakes (1 fracture, 1 osteofibrous dysplasia, 1 degenerative osteophyte and 1 schmorl node). Thus, the specificity (46.2% [6/13] vs. 69.2% [9/13]) of [^18^F]FAPI-04 PET/CT was lower than that [^18^F]FDG PET/CT (Table 2), but it was not significantly different (*P* = 0.234).

**Fig. 5:**
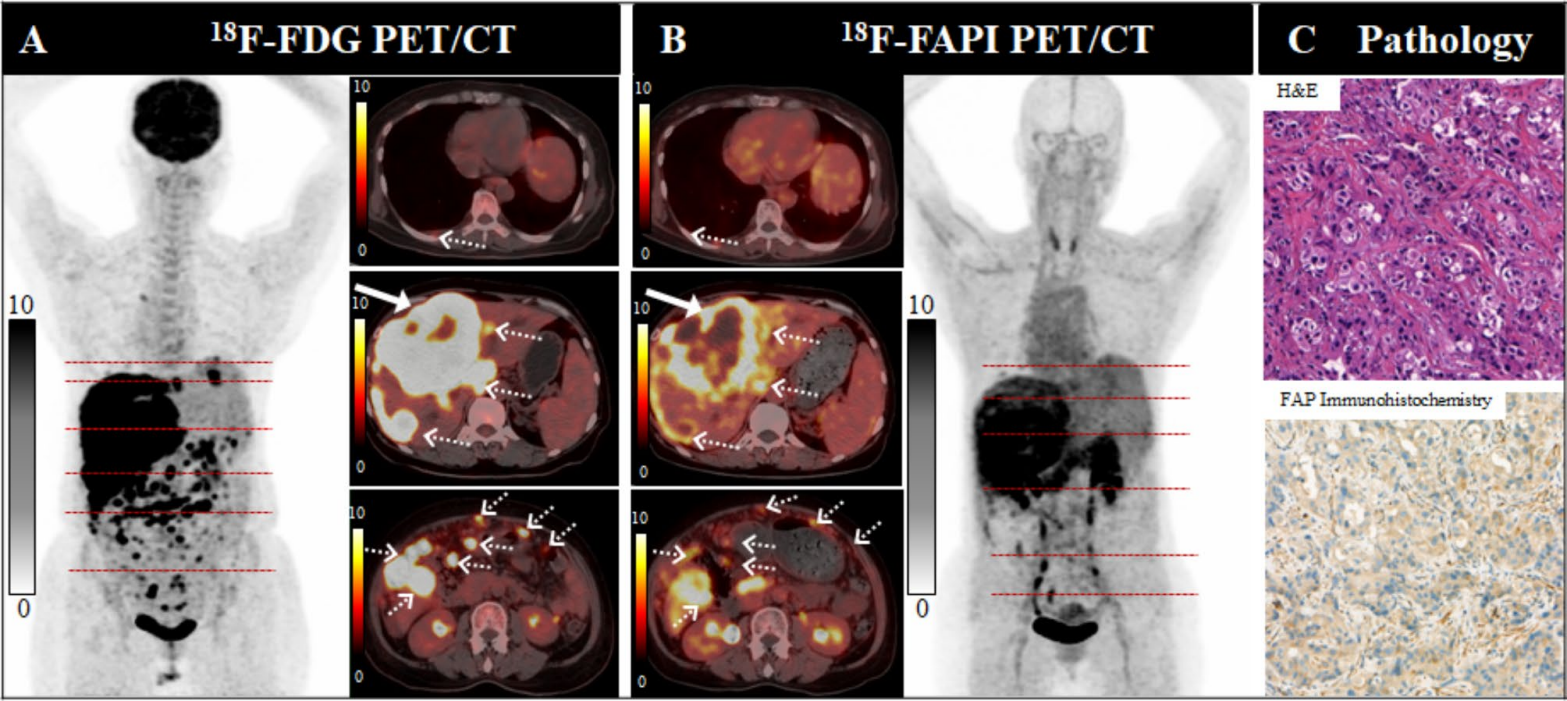
A 61-year-old female patient (Patient No. 22) with ICC was confirmed by biopsy. [^18^F]FDG PET/CT (**A**) demonstrated higher radiotracer uptake in the primary tumor (solid arrow), intrahepatic subfoci, lymph node and peritoneal metastatic lesions (dotted arrows), compared with [^18^F]FAPI-04 PET/CT (**B**). The pleural metastases exhibited a comparable radiotracer uptake to FAPI PET/CT (white arrows). Pathological findings (**C**) indicate a heightened expression for FAP

For detecting peritoneal metastases, the [^18^F]FDG PET/CT showed higher SUV_max_ than [^18^F]FAPI-04 PET/CT (median SUV_max_: 5.45 vs. 4.45, *P* = 0.003), while the [^18^F]FAPI-04 PET/CT demonstrated a larger tumor burden (median FTV vs. median MTV: 1.6 vs. 1.3, *P* = 0.025) (Table 3; Fig. 5).

### Changes in staging and therapeutic management

**Fig. 6:**
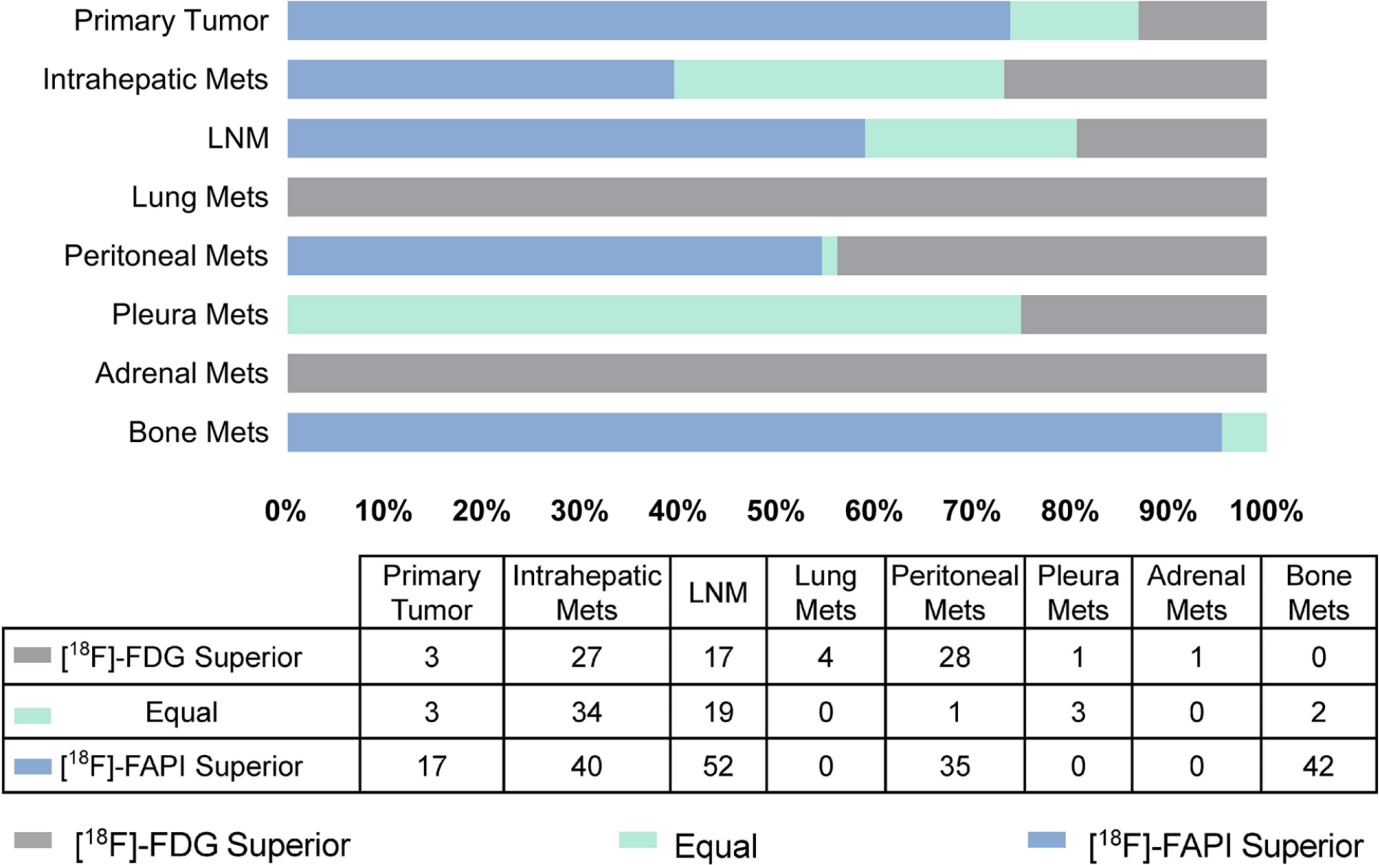
A visual comparative system is used to compare the performance of [^18^F]FDG and [^18^F]FAPI-04 PET in detecting primary tumors, intrahepatic, lymph nodes, lung, peritoneal, pleura, adrenal and bone metastases. LNM: lymph node metastases; Mets: metastases

In the initial assessment of 23 patients, [^18^F]FAPI-04 imaging detected primary ICC tumors in 3 patients with [^18^F]FDG-negative, confirmed by pathology. The patient received resection as early as possible since [^18^F]FAPI-04 detected the primary lesion. With more lymph node metastases revealed by [^18^F]FAPI-04 PET/CT than [^18^F]FDG PET/CT, the TNM staging was upgraded in 3 patients (all from II to III) (Table 4). According to the visual comparative system (Fig. 6), [^18^F]FAPI-04 PET imaging also demonstrated superior detection of primary tumors, lymph node and bone metastases compared to [^18^F]FDG PET imaging. As a result, instead of the previously planned surgical treatment for all, 3 patient received systemic chemotherapy, while one patient received palliative systemic treatment. However, [^18^F]FAPI-04 PET/CT also underestimated the TNM staging (from IV to III) in one patient due to adrenal gland metastasis detected by [^18^F]FDG, but the planning therapy was not change. Finally, TNM staging was upgraded in 6 patients (6/23, 26.1%) and downgraded in one patient, leading to changes in the planned therapy for 6 patients.

**Table 4:** Comparison of [^18^F]FDG PET/CT and [^18^F]FAPI-04 PET/CT-based TNM staging of 23 treatment-naive patients with ICC

No	TNM stage (FDG-based)	TNM stage (FAPI-based)	Additional finding on FAPI PET/CT (compared to FDG)	Staging change (compared to FDG)
Patient 1	IV	IV	LN (abdomen) mets detected	None
Patient 2	0	II	PT detected	Upstaged
Patient 3	IV	IV	More LN mets detected by FDG PET	None
Patient 4	0	IIIB	PT detected	Upstaged
Patient 5	0	II	PT and 2 intrahepatic lesions detected	Upstaged
Patient 6	II	II	More intrahepatic lesions detected	None
Patient 7	II	IIIB	LN (abdomen) mets detected	Upstaged
Patient 8	II	II	Larger disease extent of PT	None
Patient 9	II	II	Larger disease extent of PT	None
Patient 10	II	II	None	None
Patient 11*	IIIA	IIIA	None	None
Patient 12	IIIB	IIIB	None	None
Patient 13	IV	IIIB	AG mets detected by FDG PET	Understage
Patient 14	IV	IV	Greater number of bone mets detected	None
Patient 15	IIIB	IIIB	LN (mediastinal) mets detected	None
Patient 16	IV	IV	Greater number of bone mets detected	None
Patient 17	IIIB	IIIB	LN (supraclavicular) mets detected	None
Patient 18	II	II	None	None
Patient 19	IIIB	IIIB	More LN mets detected by FDG PET	None
Patient 20	II	IIIB	LN (abdomen) mets detected	Upstaged
Patient 21	II	II	None	None
Patient 22	IV	IV	Greater number of PC mets detected by FDG PET	None
Patient 23	II	IIIB	LN (abdomen) mets detected	Upstaged

*Note* The clinical stage was assigned based on American Joint Committee on Cancer (AJCC) staging system (Eighth edition)

Patient* indicates both [^18^F]FDG and [^18^F]FAPI imaging showed negative findings in lymph node metastases of ICC

*Abbreviations* PT: primary tumor, LNM: lymph node metastases, PC: peritoneal carcinomatosis, AG: Adrenal gland, Mets: metastasis

## Discussion

Accurate diagnosis and staging are crucial for the appropriate clinical management of ICC patients. [^18^F]FAPI-04 PET/CT is a novel and powerful imaging technique for the visualization of CAFs in the tumor stroma and represents a promising alternative to [^18^F]FDG PET/CT in diagnosis, initial staging, and recurrence detection for patients with liver cancer [9, 10, 16, 18, 25]. This prospective study demonstrated that [^18^F]FAPI-04 PET showed higher showed higher SUV_max_, TBR and larger tumor burden (FTV and TLF) values than [^18^F]FDG PET in the primary ICC, intrahepatic metastases, lymph node metastases, and bone metastases. Furthermore, [^18^F]FAPI-04 was superior to [^18^F]FDG in detecting primary ICC, intrahepatic metastases, lymph node metastases, peritoneal metastases and bone metastases. Compared with the TNM stage based on [^18^F]FDG, the TNM stage based on [^18^F]FAPI-04 was upgraded in 6 patients (6/23, 26.1%), resulting in management changes. Therefore, [^18^F]FAPI-04 PET/CT may be a promising techniques for diagnosis and management of ICC.

Previous studies reported great variation and low uptake of [^18^F]FDG in ICC, physiological [^18^F]FDG uptake in liver, which may lead to false-negative finding. The detection rates of ICC using [^18^F]FDG PET/CT was 70–92% [26, 27]. In the present study, the sensitivity of [^18^F]FDG PET for detecting primary ICC and intrahepatic metastases were 87% (20/23) and 76.2% (77/101), which were higher than the rate reported in previous investigations. The low-to-mild [^18^F]FDG uptake in ICC may be associated with the smaller size or higher mucin content of the tumors, low tumor-cell density, and low expression levels of glucose transporter [9, 28]. It is known that ICCs are characterized by intense stromal desmoplastic reactions surrounding cancer cells, and CAFs are the major cellular component. Previous studies demonstrated that significantly higher FAP expression on the surface of CAFs was found in ICC compared to normal liver tissue. Therefore, intense uptake of [^18^F]FAPI-04 was observed in primary ICC and intrahepatic metastases, resulting in a favorable TBR and clear tumor boundary from [^18^F]FAPI-04 PET/CT. In our study, [^18^F]FAPI-04 PET/CT detected 100% (23/23) of primary ICC, and [^18^F]FAPI-04 PET/CT showed higher SUV_max_, TBR and larger tumor burden (FTV and TLF) values than [^18^F]FDG PET/CT in the intrahepatic metastases. Similar results have been reported from some recent studies in biliary tract carcinoma [15, 19, 29].

However, in the case of obstructive cholangitis, [^18^F]FDG PET/CT showed higher radiotracer uptake in intrahepatic lesions and higher sensitivity than [^18^F]FAPI-04 PET/CT. Elevated uptake of [^18^F]FAPI-04 in tumor-associated obstructive cholangitis may mask tumor activity and increased liver background tracer uptake, then lead to false-negative finding. Additionally, focal intrahepatic cholangitis with heightened FAPI accumulation may also elevate false-positive rates, thereby reducing diagnostic specificity. The increased uptake observed in obstructive cholangitis can be explained by two primary factors. Firstly, [^18^F]FAPI is eliminated via the hepatobiliary system, leading to non-specific diffuse hepatic uptake of FAPI due to bile stasis within obstructed bile ducts [22]. Secondly, FAPI has an affinity for inflammatory cells, which may additionally stimulate a fibrotic response, thereby enhancing the avidity for FAPI [30]. Cholangitis secondary to obstruction of the proximal bile duct is a typical finding in patients with ICC. With respect to the discrimination of tumor and inflammatory lesions, we found a lower [^18^F]FAPI-04 uptake in inflammatory than in tumor lesions, although a certain overlap was observed between the uptake of [^18^F]FAPI-04 in tumor and inflammatory. In an investigation of the application of [^68^Ga]Ga-FAPI in biliary tract cancer, the study found the average SUV_max_ in inflammatory was lower than in tumor lesions [29], which was similar to our results.

Accurate evaluation of lymph node metastases is crucial for the treatment and prognosis of patients with ICC [31]. It has been reported that [^18^F]FDG PET/CT is superior to conventional image studies in detecting occult metastases in patients with invasive ICC [7]. However, [^18^F]FDG PET/CT has low sensitivity in nodal staging of ICC due to variable detection of metastatic lymph nodes and possible false-positive findings in reactive lymph nodes. In our study, the uptake and TBR of [^18^F]FAPI-04 in the positive lymph nodes were higher than those of [^18^F]FDG, and more positive lymph nodes were detected in [^18^F]FAPI-04 PET/CT than in [^18^F]FDG PET/CT. Previous studies have suggested that [^68^Ga]/[^18^F]FAPI PET may be more effective than [^18^F]FDG PET in differentiating reactive lymph nodes from tumor metastatic lymph nodes [32–34]. However, we noticed that [^18^F]FAPI-04 uptake was also demonstrated in reactive lymph nodes in our study, albeit to a lesser extent and in fewer instances compared to [^18^F]FDG PET/CT. The causes for an uptake of [^18^F]FAPI may include large or chronic inflammation accompanied by associated fibroblast activation [35]. This case indicated [^18^F]FAPI-04 maybe not a more tumor-specific imaging tracer than [^18^F]FDG for metastatic lymph node detection. Thus, it is necessary to consider false positive uptake in reactive nodes for N staging when performing [^18^F]FAPI-04 PET/CT imaging. Although differentiation of tumor and reactive lymph nodes by [^18^F]FAPI-04 PET was not the main aim of this study, this question should be investigated in future clinical trials.

Patients with ICC tends to cause bone and visceral metastases, including lung, liver, and peritoneal metastases. Considering that curative resection can sometimes be performed in patients with oligometastatic disease, early and accurate evaluation of bone and visceral metastases is important for the oncological management of ICC. In the current study, [^18^F]FAPI-04 PET/CT was superior to [^18^F]FDG PET/CT in detecting bone metastases, with higher uptake and lower background activity, consistent with results in previous studies. However, it should be noted that [^18^F]FAPI-04 PET showed lower specificity than [^18^F]FDG PET regarding bone metastases, due to more false-positive lesions being observed on [^18^F]FAPI-04 PET images. In our study, [^18^F]FAPI-04 PET showed the false-positive uptake in fractures, osteofibrous dysplasia, and degenerative osteophytes. Thus, [^18^F]FAPI-04 PET images should be interpreted cautiously to avoid misdiagnosis. Other imaging findings and clinical data should be referred to rather than solely the uptake level of [^18^F]FAPI [36]. Peritoneal metastases are another common distant metastatic pattern in ICC, and accurate assessment is crucial for selecting an appropriate therapeutic method. Previous studies demonstrated the superiority of FAPI PET/CT for detecting peritoneal metastases in various types of cancer [11, 37]. In the present study, [^18^F]FDG PET/CT missed metastases in 4 regions, although the uptake [^18^F]FDG was higher than that of [^18^F]FAPI-04 in some peritoneal metastases. The false-negative rate of [^18^F]FDG PET/CT can be relatively high because of high background tracer levels and the small size of peritoneal implants. Our data showed that all peritoneal metastases were detected by [^18^F]FAPI-04, and confirmed the usefulness of [^18^F]FAPI-04 PET/CT in the evaluation of peritoneal carcinomatosis in ICC. As a result, [^18^F]FAPI-04 PET/CT revealed more metastatic lesions than [^18^F]FDG PET/CT and led to an upgrade of the TNM stage in 26.1% of patients with ICC, especially in identifying intrahepatic metastases, lymph node metastases and bone metastases. Therefore, [^18^F]FAPI-04 PET/CT may be a promising technique for diagnosis and management of ICC.

Our study has several limitations. First of all, due to technical and ethical issues, histopathologic confirmation was not possible for all positive lesions, especially for nodal and distant metastases. Thus, in our investigation, a combination of clinical and multimodality radiographic follow-up (including abdominal ultrasound, CT/MRI and PET/CT) for more than 6 months was taken as the reference standard in our investigation. Secondly, due to the relatively rarity of ICC, the number of patients included in this study was small. Thus, the sample size needs to be increased to strengthen our conclusion in the future. Lastly, except bone and peritoneal metastases, there were few other distant metastases, such as adrenal gland, lung, and pleura metastases in this cohort to conclude the detection of those lesions. Further a well-designed prospective study involving more patients are required to explore the diagnostic and therapeutic application value of [^18^F]FAPI-04 PET/CT for patients with ICC.

## Conclusion

In this prospective study, our results exhibited that [^18^F]FAPI-04 PET/CT had higher tracer uptake than [^18^F]FDG PET/CT in intrahepatic, lymph node, and distant metastases (especially in bone) for patients with ICC. In addition, [^18^F]FAPI-04 PET/CT revealed more metastatic lesions than [^18^F]FDG PET/CT, leading to an upgrade of the TNM stage, especially in identifying intrahepatic, lymph node and bone metastases. Therefore, [^18^F]FAPI-04 PET/CT may be a promising technique in diagnostic and therapeutic management of ICC.

## Electronic supplementary material

Below is the link to the electronic supplementary material.

Supplementary Material 1

## Data Availability

The datasets used and/or analyzed during the current study are available from the corresponding author on reasonable request.

## Abbreviations

FDG: Fluorodeoxyglucose
FAPI: Fibroblast activation protein inhibitor
PET/CT: Positron emission tomography/computed tomography
ICC: Intrahepatic cholangiocarcinoma
SUV: Standardized uptake value
TBR: Tumor-to-background ratio
SUV_max_: Maximum standardized uptake value
SUV_mean_: Mean standardized uptake value
CAFs: Cancer-associated fibroblasts
FAP: Fibroblast activation protein
MTV: Metabolic tumor volume
TLG: Total lesion glycolysis
FTV: [^18^F]FAPI–avid tumor volume
TLF: Total lesion FAP expression
CT: Computed tomography
MRI: Magnetic resonance imaging
Ce-CT/MRI: Contrast-enhanced CT/MRI
TNM staging: Tumor, node, and metastasis staging
