# Supplementary material for: Role of [18F]FAPI-04 in staging and therapeutic management of intrahepatic cholangiocarcinoma: prospective comparison with [18F]FDG PET/CT
Source: EJNMMI Res. 2024 Sep 11;14:81. doi: 10.1186/s13550-024-01145-y (PMC11387567; doi:10.1186/s13550-024-01145-y)
Supplement: Supplementary file 1 — Supplementary Material 1 [file 13550_2024_1145_MOESM1_ESM.doc]

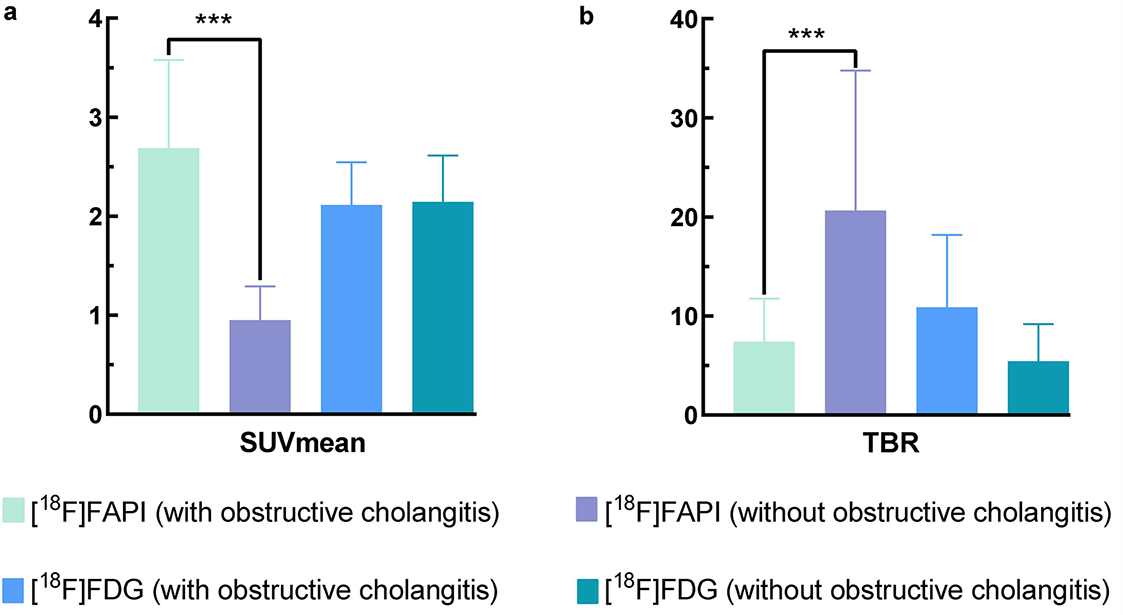


**Supplemental Figure 1.** Comparison of liver background parenchyma SUV_mean_ (a) and TBR (b) between [^18^F]FAPI-04 (LightCyan and MediumPurple) and [^18^F]FDG (DodgerBlue and

CadetBlue) PET in patients with and without obstructive cholangitis (Mann-Whitney U test). SUV_mean_ = Mean standardized uptake value; TBR = Tumor-to-background ratio; **P<0.01; ***P<0.001
